# Peptidoglycan Recognition Protein 2 Regulates Neutrophil Recruitment Into the Lungs After *Streptococcus pneumoniae* Infection

**DOI:** 10.3389/fmicb.2019.00199

**Published:** 2019-02-19

**Authors:** Alexander N. Dabrowski, Claudia Conrad, Ulrike Behrendt, Anshu Shrivastav, Nelli Baal, Sandra M. Wienhold, Holger Hackstein, Philippe D. N’Guessan, Sahar Aly, Katrin Reppe, Norbert Suttorp, Janine Zahlten

**Affiliations:** ^1^Department of Infectious Diseases and Respiratory Medicine, Charité – Universitätsmedizin Berlin, Corporate Member of Freie Universität Berlin, Humboldt-Universität zu Berlin, and Berlin Institute of Health, Berlin, Germany; ^2^Immunology and Transfusion Medicine, Justus Liebig University Giessen, Giessen, Germany; ^3^Division of Pulmonary Inflammation, Charité – Universitätsmedizin Berlin, Corporate Member of Freie Universität Berlin, Humboldt-Universität zu Berlin, and Berlin Institute of Health, Berlin, Germany

**Keywords:** infectious diseases, innate immunity, mouse, PGRP, PGLYRP2, pneumonia, *S. pneumoniae*

## Abstract

Peptidoglycan (PGN) recognition proteins (PGLYRPs) are a highly conserved group of host defense proteins in insects and mammals that sense bacterial cell wall PGN and act bactericidally or cleave PGN by amidase function. *Streptococcus* (*S.*) *pneumoniae* is one of the top five killers worldwide and causes, e.g., pneumonia, endocarditis, meningitis and sepsis. *S. pneumoniae* accounts for approximately 1.5–2 million deaths every year. The risk of antibiotic resistance and a general poor prognosis in young children and elderly people have led to the need for new treatment approaches. To the best of our knowledge, there is no report on the relevance of PGLYRP2 in lung infections. Therefore, we infected mice deficient for PGLYRP2 transnasally with *S. pneumoniae* and examined the innate immune response in comparison to WT animals. As expected, PGLYRP2-KO animals had to be sacrificed earlier than their WT counterparts, and this was due to higher bacteremia. The higher bacterial load in the PGLYRP2-KO mice was accomplished with lower amounts of proinflammatory cytokines in the lungs. This led to an abolished recruitment of neutrophils into the lungs, the spread of bacteria and the subsequent aggravated course of the disease and early mortality of the PGLYRP2-KO mice. These data suggest a substantial role of PGLYRP2 in the early defense against *S. pneumoniae* infection, and PGLYRP2 might also affect other infections in the lungs.

## Introduction

Pneumonia is a common infectious disease divided into several environmental classes, including hospital-associated pneumonia (hospital-acquired pneumonia, ventilator-associated pneumonia, healthcare-associated pneumonia) and community-acquired pneumonia (CAP). Whereas hospital-associated pneumonia occurs during or after hospitalization or treatment of generally sick and/or immunosuppressed patients, CAP occurs outside the hospital in all kinds of people (Anand and Kollef, 2009). Therefore, the common pathogens and the course of pneumonia differ in these groups. In CAP, there is a u-shaped curve of mortality with the highest rates in individuals below 5 and higher than 65 years of age and a very low overall mortality (below 1% of all cases) (Mandell, 2015; Prina et al., 2015). Nevertheless, CAP is one of the main causes of death worldwide, and in patients who need to be hospitalized, short-term mortality of up to 50% with an estimated overall mortality of approximately 14% is reported (Mandell, 2015; Prina et al., 2015). In up to 50% of all cases of CAP, *Streptococcus* (*S.*) *pneumoniae*—also called pneumococcus—is the main causative pathogen for this disease, which costs approximately 1.5–2 million lives every year (Höffken et al., 2010; Dockrell et al., 2012; Troeger et al., 2017).

*S. pneumoniae* is a gram-positive diplococcus that resides asymptomatically in the nasopharynx of many healthy individuals. In susceptible individuals, *S. pneumoniae* cannot only lead to pneumonia but also spread from the respiratory tract into the blood and distal organs and can cause, e.g., sepsis, meningitis, rhinitis, sinusitis, and endocarditis (Bhatty et al., 2011; Geno et al., 2015). Currently, 97 different serotypes of pneumococci are known, characterized by their different polysaccharide capsules (Geno et al., 2015). The capsule is, on the one hand, a major virulence factor that protects pneumococcal cell wall components, such as peptidoglycan (PGN) and (lipo-) teichoic acids, from recognition by the immune system via pattern recognition receptors (PRRs) or the complement system and degraded by host defense molecules (HDMs) (Geno et al., 2015). On the other hand, the capsule can hinder bacteria, e.g., from traversing the epithelial barrier and entering the blood stream (Hammerschmidt et al., 2005).

Peptidoglycan recognition proteins (PGRPs) are a class of HDMs that were first described in 1996 independently by two groups. Yoshida et al. (1996). isolated a PGRP from the silkworm (*Bombyx mori*). Kustikova et al. (1996) found the tag7 protein (also known as PGRP-S or PGLYRP1) in mice. In insects, there is a wide variety of different PGRPs. The anopheles or the drosophila for example have 9 and more than 19 different proteins/splice variants of this group, respectively. However, in mammals there are only four known proteins and named as PGLYRP1 through PGLYRP4 (Kurata, 2014; Dziarski and Gupta, 2018).

For human PGLYRPs 1, 3, and 4, a direct bacteriostatic or bactericidal effect has been shown for more than a decade (Lu et al., 2006), but PGLYRP2 was mostly considered to function as an amidase by hydrolyzing bacterial cell wall PGN (Gelius et al., 2003; Wang et al., 2003). Additionally, the influences of PGLYRP2 on innate immune signaling have been discussed, as PGLYRP2 might change the recognition of PGN via NOD1/NOD2 receptors by degradation (Chaput et al., 2013). Recently, reports have shown direct antibacterial functions for PGLYRP2 (Bobrovsky et al., 2016; Torrens et al., 2017). Furthermore, PGLYRPs play a role in the maintenance of normal microbiota (Saha et al., 2010). Gene knockouts for one of the four genes lead to a more proinflammatory phenotype of the microbiome, which could lead to more severe disease outcomes (Saha et al., 2010; Dziarski et al., 2016).

Other functions of PGLYRPs include anti-tumor and chemotactic activities for PGLYRP1 (Dukhanina et al., 2015; Sharapova et al., 2017a,b), modulation of phagocytosis and cytokine release for PGLYRP3 (Zenhom et al., 2011; De Marzi et al., 2015) and regulation of the Treg/Th17 balance for PGLYRP4 (Park et al., 2011b). The different abilities to function as an antibacterial substance or an amidase or immune sensor, are likely due to different binding sites and a highly variable *N*-terminal region of the protein structure (Guan et al., 2005). PGLYRPs have two different binding sites, which are opposite of each other. First, there is a PGN binding site, which binds degradation products of PGN, and second, there is an alternative binding site, which may function as a mediator of immune receptors (Guan et al., 2005).

These functions might lead to different regulatory mechanisms, which could have opposing effects for gene knockouts of different PGLYRPs in a single disease or in the knockout of a single PGLYRP in different diseases. For example, when infecting the murine cornea with *Pseudomonas aeruginosa* to induce corneal keratitis, it was illustrated that PGLYRP2-KO mice had better clearance and lower clinical scores (Gowda et al., 2015). Furthermore, these mice were nearly fully protected against PGN- or muramyl dipeptide (MDP)-induced arthritis (Saha et al., 2009). On the other hand, PGLYRP2-KO mice are more susceptible to chemically induced psoriasis-like skin inflammation (Park et al., 2011a) or DSS-induced colitis (Saha et al., 2010). However, reports of activity against pneumococci are rare. There is only one report for PGLYRP3 (Shrivastav et al., 2018), showing no effect on *S. pneumoniae* lung infection in mice. Furthermore, unpublished observations by our group show indirect immunomodulatory effects by PGLYRP4 in the same experimental setting.

Understanding the mechanisms of endogenous HDMs could lead to new and innovative options to treat antibiotic-resistant microbes. Therefore, we aimed to elucidate the influences of PGLYRP2 in pneumococcal pneumonia. This disease is a major cause of death, especially in people with lower functioning immune systems, such as young children and elderly people. Here, to the best of our knowledge, we are the first to report the direct impact of the PGLYRP2 gene knockout on bacterial lung infection and to illustrate that PGLYRP2 is important for host defense. We further analyzed changes in the innate immune system and demonstrated important new insights into the regulation of cell recruitment into the lungs by the host defense molecule PGLYRP2.

## Materials and Methods

### Animals

Prof. Dr. Roman Dziarski (Department of Microbiology and Immunology, Indiana University School of Medicine, Indiana, United States) kindly provided the breeding pairs for the PGLYRP2-KO and WT mice. Animals were generated as described before on a BALB/c background (Saha et al., 2009). Mice were bred and housed at the central breeding facility of the Charité–Universitätsmedizin Berlin (Forschungseinrichtung für Experimentelle Medizin, FEM) under specific pathogen-free conditions. All experimental procedures were in compliance with the Federation of European Laboratory Animal Science Associations (FELASA) guidelines and recommendations for the care and use of laboratory animals, as well as approved by local institutional (Charité-Universitätsmedizin Berlin) and governmental (Landesamt für Gesundheit und Soziales Berlin, approval ID: G0220/13) authorities. Animals were housed at a 12 h light/dark cycle, with food and water *ad libitum*. In all experiments, the mice were anesthetized intraperitoneally (i.p.) with ketamine and xylazine, and their suffering was minimized in compliance with the 3R principles.

### Genotyping

Mice were routinely analyzed for the genotype as described previously (Saha et al., 2009; Shrivastav et al., 2018). Briefly, tail tips were digested overnight in Tris-EDTA-SDS buffer and Proteinase K (Sigma-Aldrich, St. Louis, MO, United States). DNA was isolated to perform PCR with the following primers: GCTGCGCAATTCGCGCAGGTCTC (WT, forward) and ATGGTTCCATCAGCAAAGTGCTGG (WT, reverse); TGCGAGGCCAGAGGCCACTTGTGTAGC (PGLYRP2, forward) and ATGGTTCCATCAGCAAAGTGCTGG (PGLYRP2, reverse). DreamTaq DNA Polymerase was used according to the manufacturer’s instructions (Life Technologies GmbH; Darmstadt; Germany). PCR products were run on an agarose gel, and DNA bands were visualized with ethidium bromide under UV light.

### Bacteria

We used the *S. pneumoniae* WT serotype 3 strain A66 (NCTC 7978) for all experiments. Streptococci were grown on Columbia agar with 5% sheep blood (BD Biosciences, Heidelberg, Germany) overnight (37°C, 5% CO_2_). Single colonies were picked and inoculated in Todd-Hewitt broth + 0.5% yeast (both BD Biosciences) supplemented with 10% FCS. Bacteria were grown until the mid-log phase (OD_600_: 0.3-0.4, 37°C, 5% CO_2_), centrifuged (2,000 × *g*, 10 min) and resuspended in cell culture media (*in vitro* stimulation of cells) or sterile PBS (*in vivo* infection) at the appropriate concentration.

### Mouse Infection Model

Female BALB/c WT or BALB/c PGLYRP2-KO mice (8-12 weeks) were anesthetized by i.p. injection of ketamine and xylazine (both Rotexmedica, Luitré, France) and inoculated transnasally with 20 μl of bacterial suspension of *S. pneumoniae* or 20 μl of sterile PBS as a control. In total, 98 mice were used in this study: 22 mice for bacterial load (11 mice per group), 49 mice for approximate survival (25 WT and 24 PGLYRP2-KO mice) and 27 mice for cell recruitment and cytokine measurements (each 6 uninfected WT and PGLYRP2-KO, 8 infected WT and 7 infected PGLYRP2-KO mice). Mice were monitored before infection and every 12 h thereafter to assess clinical signs of illness (blowsy fur, crusty secretion on the eye rim, reduced reaction or movement, isolation, temperature ≤ 35.0°C, body weight loss, accelerated or cumbersome breathing, pain, paleness, staggering). For the evaluation of the approximate survival after pneumococcal infection, disease severity was monitored for 10 days. Mice were euthanized by cervical dislocation after i.p. injection of ketamine and xylazine when they reached at least one of the predefined humane endpoints or at the end of the 10 days period. Humane endpoints were defined as (i) body weight loss ≥ 25%, (ii) cumbersome breathing, and (iii) accelerated breathing in combination with staggering or pain.

### Bacterial Load

Lung and spleen homogenates or EDTA blood was plated on Columbia agar with 5% sheep blood in serial dilutions. After overnight incubation at 37°C with 5% CO_2_, the colonies were counted manually, and the CFUs were calculated.

### Cell Isolation

Primary cells from untreated WT mice were isolated after anesthesia with ketamine and xylazine and exsanguination by opening the *vena cava caudalis*. In the case of alveolar epithelial cells, heparin (Rotexmedica) was included in the anesthesia mix.

#### Alveolar Macrophages (AMs)

AMs were isolated as described earlier (Cakarova et al., 2009). Briefly, the lungs were lavaged (2 mM EDTA in PBS, 5 ml), and the cell suspension centrifuged (300 × *g*, 10 min, 4°C). Afterward, the cells were suspended in RPMI1640 (10% FCS, 1% Glu, 1% P/S) and seeded in cell culture plates. The medium was exchanged 2 h later to remove non-adherent cells. Cell purity and morphology was routinely checked by light microscopy. The next day, the medium was changed to RPMI1640 (2% FCS, 1% Glu) 2 h before infection.

#### Alveolar Epithelial Cells (AECs)

AECs were harvested from perfused and digested lungs (5,000 U dispase) as described previously (Cakarova et al., 2009) with some modifications as follows: after generation of the single cell suspension by passing macerated lungs through different cell strainers (100, 70, and 30 μm), the suspension was centrifuged (200 × *g*, 10 min) and resuspended in PBS containing 3% FCS and 10 mM EDTA. Unwanted cells were depleted by the addition of biotinylated anti-CD45, anti-CD16/CD32, anti-CD31 antibodies (BD Biosciences) and MagniSort Streptavidin Negative Selection Beads (eBioscience, Frankfurt am Main, Germany). Then, cells were seeded in DMEM (10% FCS, 1% Glu, 1% P/S) in cell culture plates and incubated (37°C, 5% CO_2_, overnight). Cell morphology was routinely checked by light microscopy. In addition, FACS staining was performed for cell purity and was always above 90% (see also Cakarova et al., 2009). Two hours before the experiment, the medium was changed to DMEM containing 2% FCS and 1% Glu.

#### Bone Marrow Derived Neutrophils (PMNs)

For isolating PMNs, the femurs and tibiae of mice were flushed with PBS, and cells were filtered through a 70 μm cell strainer as described earlier (Shrivastav et al., 2018). PMNs were then positively isolated using the mouse anti-Ly6G MicroBead kit (Milteny Biotec GmbH, Telterow, Germany) according to manufacturer’s instruction, seeded in RPMI1640 containing 2% FCS and 1% Glu and infected immediately. This cell isolation method is considered to provide highly pure and viable cells (Swamydas et al., 2015). Furthermore, the positive selected PMNs can strongly induced proinflammatory cytokines (as assed by ELISA, data not shown), indicating that these cells have not lost their capacity to respond to bacteria.

### Gene Expression Analysis

Analysis of the gene expression of *Pglyrp2* was performed in isolated primary BALB/c WT AECs, AMs, and PMNs that were stimulated for 6 h with *S. pneumoniae*. RNA was extracted from the cells using TRIzol (Thermo Fisher Scientific, Schwerte, Germany) according to the manufacturer’s instructions. Total RNA (1 μg) was used to transcribe cDNA (High-Capacity cDNA Reverse Transcription Kit; Life Technologies GmbH). Samples were preamplified for 18 cycles (TaqManPreAmp Master Mix Kit; Life Technologies GmbH) and used for TaqMan^®^ quantitative gene expression analysis using specific primers (Taqman assay sequence numbers: *Gapdh* Mm99999915_g1, *Pglyrp2* Mm01348077_m1; both Life Technologies GmbH). Quantitative real-time PCR was performed according to the manufacturer’s instructions using the following cycling conditions: 2 min, 50°C; 10 min, 95°C and 40 cycles with 15 s, 95°C and 1 min, 60°C. The efficiency-corrected ΔΔ*C_T_* method was used for the calculation of the relative expression (Pfaffl, 2001). *Gapdh* was used as a housekeeping gene, and uninfected WT cells were used as an untreated control.

### Cytokine Measurement

Mice infected with *S. pneumoniae* were sacrificed 48 h post-infection (hpi), and the lungs were taken and homogenized in HBSS with a protease inhibitor (Complete^®^ Mini, Roche, Mannheim, Germany) in a FastPrep-24 (MP Biomedicals, Eschwege, Germany). The homogenate was centrifuged at 14,000 × *g* for 10 min, and the supernatant was analyzed by a mouse multiplex ELISA according to the manufacturer’s instructions (Procartaplex, eBiosience).

### Cell Recruitment

Lungs from mice infected with *S. pneumoniae* or PBS-instilled control mice were processed according to Shrivastav et al. (2018). Briefly, lungs were perfused via the heart with HBSS, minced in RPMI1640 with 10% FCS and digested with Collagenase A and DNase I (both Roche) at 37°C for 1 h. A single cell suspension was generated, washed with HBSS and blocked with mouse serum.

Differentially conjugated monoclonal antibodies against the following markers, as well as the appropriate isotype controls, were used for surface staining: CD3e, CD4, CD8a, CD11b, CD11c, CD19, CD45, CD49b, F4/80, TCR-γδ, GR1, I-A/I-E, CD64, and SiglecF (BioLegend, Aachen, Germany). Cells were washed with HBSS, fixed with 2% PFA (Merck, Darmstadt, Germany), washed again, suspended in HBSS and kept at 4°C until measurement. Flow cytometry was performed on a FACS ARIA III flow cytometer (BD Biosciences), with 100,000 events per sample at a minimum.

Neutrophils were identified as CD45^+^, GR1^high^, CD11b^high^, AMs as CD45^+^, CD11c^high^, SiglecF^high^ and dendritic cells (DCs) as CD45^+^, CD11c^high^, SiglecF^-^/^low^, CD49b^-^, MHC II^high/int^. Classical B cells were identified as CD45^+^, CD3^-^, CD49b^-^, CD19^+^, whereas classical T cell populations were divided into T helper cells (CD45^+^, CD3^+^, CD4^+^, CD8^-^) and cytotoxic T cells (CD45^+^, CD3^+^, CD8^+^, CD4^-^). Furthermore, we stained for the two major innate lymphocyte subsets γδ T cells (CD45^+^, CD3^+^, CD4^-^, CD8^-^, TCR-γδ^+^) and NK cells (CD45^+^, CD3^+^, CD49b^+^, CD19^-^). The classical NK cell marker NK1.1 is not expressed in BALB/c mice. Therefore, we used the pan-NK cell marker CD49b (Hackstein et al., 2012).

### Data Analysis

Analysis of flow cytometry data was performed using FlowJo v10 (FlowJo, LLC, Ashland, OR, United States). Statistical analysis was performed using Prism 6 (GraphPad software, La Jolla, CA, United States). Samples were categorized by optical analysis and Shapiro-Wilkinson normality test (threshold at *p* = 0.05) to one of the following distributions: (A) Gaussian, (B) log-normal, (C) other non-Gaussian. Statistical significance (*p* ≤ 0.05) was then analyzed by appropriate tests as stated in the respective figure legends. Differences in survival were analyzed by the Gehan-Breslow-Wilcoxon test. Significance is indicated with asterisks (^∗^) if *p* ≤ 0.05. The exact *p*-values are provided if *p* < 0.1, but >0.05 and the results are considered not significant if *p* ≥ 0.1.

## Results

### Gene Expression of *Pglyrp2* Was Induced in Bone Marrow-Derived Neutrophils and Macrophages

**FIGURE 1:**
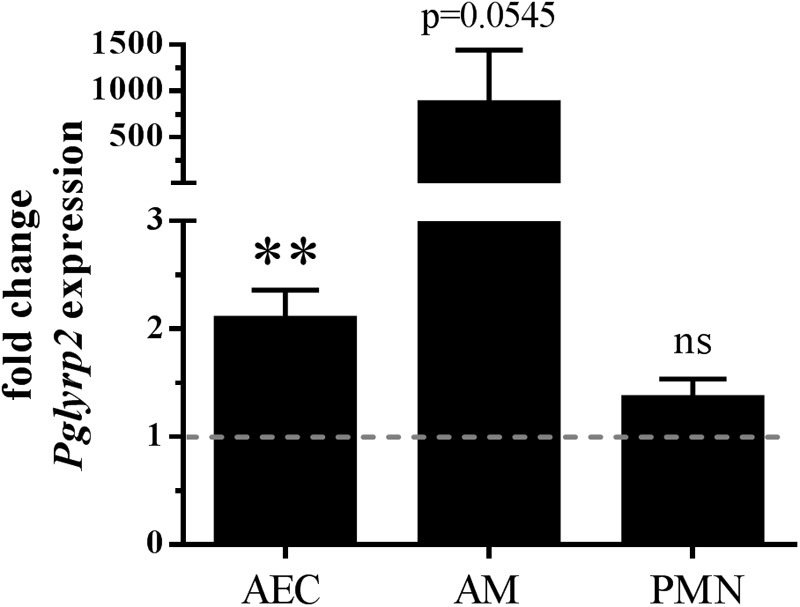
Enhanced gene expression of *Pglyrp2* in isolated primary BALB/c WT cells induced by *S. pneumoniae*. Alveolar epithelial cells (AECs), alveolar macrophages (AMs) and bone marrow-derived neutrophils (PMNs) were infected for 6 h with *S. pneumoniae* (A66) to analyze the mRNA expression of *Pglyrp2* by qPCR. Relative expression was calculated by the ΔΔ*C_T_* method with *Gapdh* as the housekeeping gene and uninfected WT cells as the control. Three (AECs, PMNs) or four (AMs) independent experiments (mean + SEM). The dotted line represents the level of untreated control. Statistics: Student’s *t-*test on logarithmic data. ^∗∗^*p* ≤ 0.01, ns, not significant.

There is little knowledge about the expression of PGLYRPs in lungs. Only single reports showed expression of PGLYRP1 in mouse lungs at the mRNA and protein levels (Yao et al., 2013) or *Pglyrp3* and *Pglyrp4* in mouse lungs at the mRNA level (Mathur et al., 2004). Furthermore, we recently showed that *Pglyrp3* is expressed in AMs, AECs, and PMNs (Shrivastav et al., 2018). Because we are especially interested in resident and recruited cells sensing or fighting *S. pneumoniae* in the lungs, we started our examinations by analyzing the expression of *Pglyrp2* in uninfected and infected isolates of primary BALB/c WT cells. We chose to analyze AECs, AMs, and PMNs because these cells are either the first cells to recognize invading pathogens (AECs and AMs) or are the major cell type for clearance of *S. pneumoniae* (PMNs) (Kolaczkowska and Kubes, 2013; Hallstrand et al., 2014). *Pglyrp2* mRNA was detectable at very low levels in PMNs, AMs and AECs. *In vitro* infection of these primary cells with *S. pneumoniae* significantly induced *Pglyrp2* expression in AECs by twofold and by approximately 850-fold in AMs but did not alter the expression in PMNs (Figure 1).

### PGLYRP2-KO Mice Show an Aggravated Course of Bacterial Pneumonia Compared to WT Mice

**FIGURE 2:**
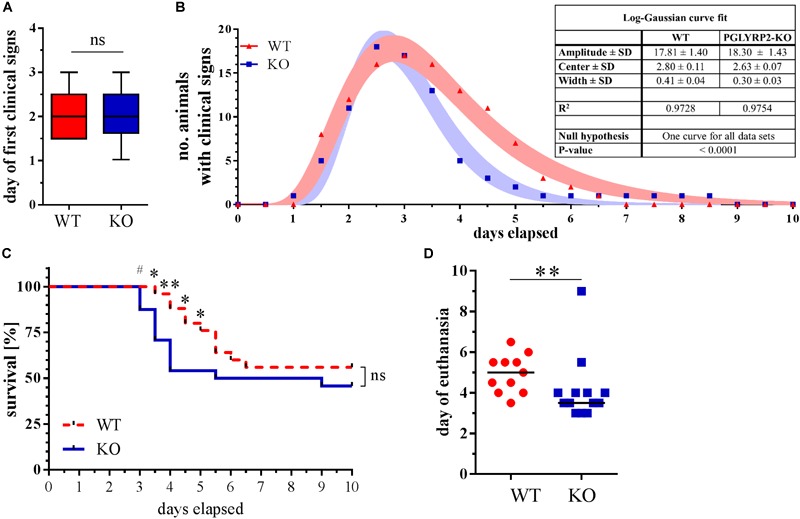
Earlier death of PGLYRP2-KO mice in a 10-day approximate survival analysis of infected mice. PGLYRP2-KO and WT mice were infected with *S. pneumoniae* (A66, 5 × 10^4^ CFU). Clinical signs and survival were recorded for a period of 10 days every 12 h (WT: *n* = 25, PGLYRP2-KO: *n* = 24; five independent experiments). **(A)** The day the first clinical signs were observed is given as the median ± 95% CI and **(B)** the number of sick animals is expressed as total numbers without dead animals. Dots represent the number of sick animals, and bands represent the 95% CI of a log-Gaussian curve fit. The number of animals that showed clinical signs of sickness and are therefore included in this analysis are *n* = 17 for WT and *n* = 20 for PGLYRP2-KO. **(C)** Survival is shown as the Kaplan-Mayer curve, and **(D)** the day of death was assessed by excluding all surviving animals and calculating the median and 95% confidence interval from the deceased animals (WT: *n* = 11, PGLYRP2-KO: *n* = 13). Statistics: **(A,D)** Mann–Whitney *U*-test and **(B)** log-Gaussian fit, **(C)** Gehan-Breslow-Wilcoxon test. ^#^*p* ≤ 0.1, ^∗^*p* ≤ 0.05, ^∗∗^*p* ≤ 0.01, ns, not significant.

Deletion of PGLYRP2 might lead to an altered immune response to pathogens due to differences in the sensing of PGNs or direct antibacterial activity. Therefore, we first analyzed the course and severity of pneumococcal pneumonia with an approximate survival analysis. After infection, WT and PGLYRP2-KO mice showed clinical manifestation of the infection in a mean of 2 days (Figure 2A). While the first occurrence of clinical signs was visible 1 day after infection, most animals were euthanized according to the predefined endpoints or recovered after 5–6 days (Figure 2B). The course of clinical apparent pneumonia was significantly different in the WT vs. PGLYRP2-KO mice (*p* < 0.0001, Figure 2B). The first mice reached the predefined humane endpoints for euthanasia 3 days after infection and there were significant differences in the approximate survival between days 3.5 and 5 in the PGLYRP2-KO vs. WT mice, but not at the end of the experiment (Figure 2C). Nevertheless, there was a significant difference in the time when mice had to be sacrificed in the PGLYRP2-KO and WT mice (Figure 2D). The PGLYRP2-KO mice had to be euthanized at a median of 1.5 days earlier (median: 3.5 days, interquartile range (IQR): 3.25-4.0) than WT animals (median: 5.0 days, IQR: 4.0-5.5). The faster progression of pneumococcal disease in the PGLYRP2-KO animals is not only represented by the approximate survival curve but also by the clinical score. The PGLYRP2-KO mice showed significantly higher clinical scores compared to the WT mice at 3 days and higher scores 3.5 days p.i., whereas the WT mice showed elevated clinical scores for a longer period of time (Supplementary Figures S1A,B).

### PGLYRP2-KO Mice Have a Higher Susceptibility for Bacterial Spread Into the Periphery

**FIGURE 3:**
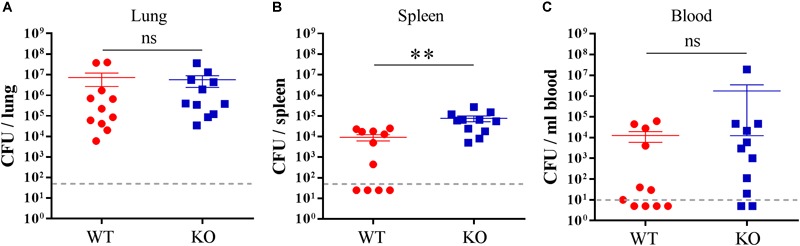
Higher bacterial burden in the spleen of the PGLYRP2-KO vs. WT mice. Bacterial burden was determined in the **(A)** lung, **(B)** spleen, and **(C)** blood of *S. pneumoniae*-infected (A66, 10^5^ CFU) animals 48 hpi. The results are from 11 animals per group in three independent experiments. Colony counts are expressed as the mean ± SEM. The dotted line represents the lower limit of detection. Undetected samples were set to half of the LLOD for statistical purposes. Statistics: Student’s *t*-test on logarithmic data. ^∗∗^*p* ≤ 0.01, ns, not significant.

Next, we wanted to investigate whether PGLYRP2 deficiency leads to an altered restriction of bacterial growth upon infection with *S. pneumoniae.* We chose the time when mice first showed clinical signs of illness and analyzed the bacterial burden in the lungs, spleens and blood. While there was no difference in the bacterial burden in lungs (Figure 3A), there were significantly more bacteria in the spleens (Figure 3B) and a trend toward higher bacterial burden in the blood (Figure 3C) in the PGLYRP2-KO compared to the WT mice.

### Lower Amounts of Neutrophil Attracting KC and Proinflammatory Cytokines Were Detected in Lungs of the Infected PGLYRP2-KO Than the WT Mice

**FIGURE 4:**
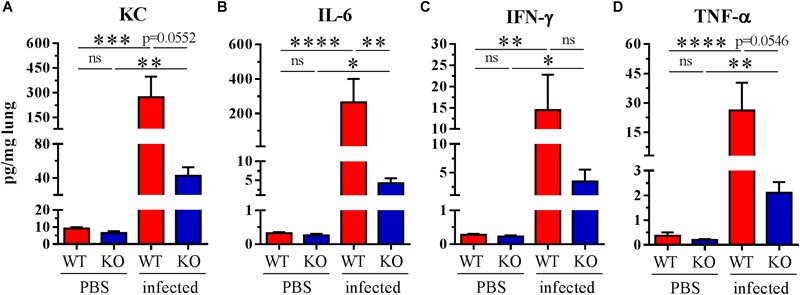
Impaired cytokine secretion in whole lung homogenates of PGLYRP2-KO mice. Mice were infected with *S. pneumoniae* (A66, 10^5^ CFU) for 48 h. **(A)** KC, **(B)** IL-6, **(C)** IFN-γ, and **(D)** TNF-α were measured by multiplex ELISA. Sample size: WT and PGLYRP2-KO PBS: *n* = 6 each, WT infected: *n* = 8, PGLYRP2-KO infected: *n* = 7. Data are represented by the means + SEMs. Thresholds for nondetection were set to the half of the lower limit of detection for statistical purposes. Statistics: ordinary one-way ANOVA with Holm-Sidak’s correction for multiple comparisons on logarithmic data. ^∗^*p* ≤ 0.05, ^∗∗^*p* ≤ 0.01, ^∗∗∗^*p* ≤ 0.001, ^∗∗∗∗^*p* ≤ 0.0001, ns: not significant.

To check if there was a lower proinflammatory response in the PGLYRP2-KO mice, the *in vivo* cytokine response to *S. pneumoniae* infection in the lungs was analyzed 48 hpi. Indeed, there was less of the proinflammatory and neutrophil attracting chemokine KC (a mouse homolog to human IL-8) (Figure 4A) and significantly less proinflammatory IL-6 (Figure 4B) detectable in the lung homogenates of PGLYRP2-KO compared to WT mice. In addition, we observed lower amounts of IFN-γ and TNF-α (Figure 4C,D) in PGLYRP2-KO mice. In conclusion, there is a reduction in cytokine level in lungs from infected PGLYRP2-KO vs. WT mice although there were no significant differences in the bacterial load at this time point (Figure 3A).

### Lower Number of Neutrophils Were Recruited to the Site of Infection 48 hpi

**FIGURE 5:**
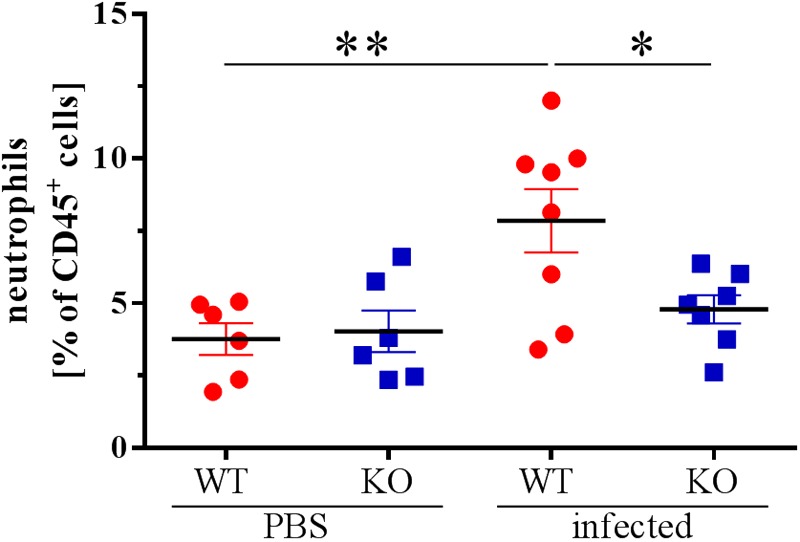
Reduced recruitment of neutrophils into the lungs of PGLYRP2-KO mice 48 hpi. Recruitment of PMNs into the lungs was analyzed by flow cytometry in PBS and *S. pneumoniae*-infected animals 48 hpi (A66, 10^5^ CFU). Means ± SEMs of six (WT PBS and PGLYRP2-KO PBS), seven (PGLYRP2-KO infected) or eight (WT infected) mice. Statistics: ordinary one-way ANOVA with Holm-Sidak’s correction for multiple comparisons. ^∗^*p* ≤ 0.05, ^∗∗^*p* ≤ 0.01, ns, not significant.

Because alterations in the cytokine profile can change immune responses, e.g., cell recruitment, we analyzed the lungs of infected and uninfected animals 48 hpi for the proportion of different immune cells. Strikingly, we could not detect a recruitment of neutrophils into the lungs of the PGLYRP2-KO animals 48 hpi, whereas WT animals significantly recruited neutrophils into the lungs (Figure 5). However, no effects were seen on the total leukocyte count (Supplementary Figure S2A) or in other major innate cell populations such as AMs (Supplementary Figure S2B), DCs (Supplementary Figure S2C), γδ T cells (Supplementary Figure S2D) and NK cells (Supplementary Figure S2E). B cells were reduced in infected WT and PGLYRP2-KO mice compared to the PBS-treated mice, but no difference could be detected between the WT and PGLYRP2-KO mice (Supplementary Figure S2F). The PGLYRP2-KO mice had lower basal levels of Tc cells (Supplementary Figure S2G), but their Th cells (Supplementary Figure S2H) did not show any difference at 48 hpi in the infectious state.

## Discussion

Host defense molecules have a major impact on the defense of invading microorganisms. They can act through direct and indirect mechanisms such as direct killing or arresting of growth and replication or by activating innate and adaptive immune cells to let them sense, kill and clear the unwanted microorganisms (Hancock et al., 2016). If these mechanisms are altered or not fully mature, invading pathogens such as *S. pneumoniae* can evade sensing and killing and lead to severe life-threatening diseases. We commonly treat such bacterial infections with antibiotics, but, e.g., emerging resistance makes treatment difficult and too often inefficient (José et al., 2015; Spellberg et al., 2015). Understanding the mechanisms of endogenous HDMs could lead to new approaches to cure life-threatening diseases. All four mammalian PGLYRPs are known for their antibacterial functions (Lu et al., 2006; Wang et al., 2007; Bobrovsky et al., 2016; Torrens et al., 2017), and PGLYRPs 1 and 3 were shown to also have immune modulatory functions (Zenhom et al., 2011; De Marzi et al., 2015; Sharapova et al., 2017b).

To fulfill these important functions, HDMs need to be expressed in the same areas where pathogens and commensals reside or they need to be transported to these locations. It is known that PGLYRPs 3 and 4 are expressed, e.g., in epithelial cells from the gut to the skin, and the eye to the lungs (Liu et al., 2001; Mathur et al., 2004; Uehara et al., 2007; Gowda et al., 2015; Hua et al., 2015; Shrivastav et al., 2018). PGLYRP1 is mainly expressed and stored in the granules of PMNs and may therefore act directly on pathogens or on cells in the proximity of pathogens after degranulation of the PMNs (Liu et al., 2000). Nevertheless, PGLYRP1 is also expressed in diverse epithelial cells and in macrophages in the lung (Yao et al., 2013). The fourth member of this class of HDMs, PGLYRP2, is mainly expressed in the liver and secreted into the blood (Xu et al., 2004). Its expression was also determined, e.g., in the intestine (Duerr et al., 2011; Lee et al., 2012), in keratinocytes (Wang et al., 2005), in the brain (Arentsen et al., 2017), as well as in the lungs of monkeys (Maniar-Hew et al., 2013). Own unpublished observations in C57BL/6 mice indicated, that the expression of *Pglyrp2* is decreased in whole lung tissue during pneumococcal infection. However, so far, it is unknown which cells in the lung express PGLYRP2. We showed here for the first time that AECs and AMs express *Pglyrp2* and that this expression is upregulated in both cell types upon infection with *S. pneumoniae*. This is contrary to our previous unpublished observations in C57BL/6 mice, but indicate, that there might be different expression patterns ins different mouse modes, *in vivo* and *in vitro* infection, or due to blood cells, which were still in the lung during RNA isolation. AECs and AMs are the first cells to come in close contact with invading pathogens in the lower respiratory tract and these cells are important for, e.g., the establishment of the barrier function and the recruitment and activation of PMNs (Kolaczkowska and Kubes, 2013; Hallstrand et al., 2014). This led us to hypothesize that there might be a difference in the local lung environment leading to a change in bacterial clearance.

Therefore, we analyzed the bacterial burden of infected animals and found no differences in lungs of PGLYRP2-KO and WT mice. In contrast, there were significantly more bacteria in the spleens of the PGLYRP2-KO vs. WT mice and the PGLYRP2-KO mice also tended to have more bacteria in their blood. Therefore, it seemed that bacteria can more easily disseminate into the periphery of the PGLYRP2-KO mice. A greater spread of bacteria can be accompanied by a reduced control of immunity by lower levels of proinflammatory cytokine release in the lungs and lower recruitment of e.g., phagocytic immune cells. This phenotype was already described for the PGLYRP2-KO mice in a MDP-induced arthritis model. The authors showed a reduced production of local proinflammatory cytokines and a reduced recruitment of PMNs into the hind limbs of the PGLYRP2-KO vs. WT mice (Saha et al., 2009). Therefore, we analyzed the local proinflammatory cytokine response and could indeed show lower amounts of KC, IL-6 and TNF-α. KC especially is strongly associated with the recruitment of neutrophils but TNF-α is also known to have functions in adherence and trafficking of PMNs (Kolaczkowska and Kubes, 2013; Rossaint and Zarbock, 2013). IFN-γ and IL-6, however, have no chemotactic effects (Borish et al., 1989; Ellis and Beaman, 2004), but they are important for the activation of PMNs (Borish et al., 1989; Ellis and Beaman, 2004; Kolaczkowska and Kubes, 2013; Rossaint and Zarbock, 2013).

Thus far, the mechanism behind this reduced cytokine response is still unclear. One possibility is an alteration of *S. pneumoniae* PGN detection. However, Saha et al. (2009) reported that the effects of PGLYRP2 on MDP-induced arthritis is independent of the amidase activity. Another mechanism could be an influence on the NF-κB pathway. Zenhom et al. described a regulation of IκBα by PGLYRP3. Other mechanisms could be imagined and should be addressed in the future.

Because of this reduced proinflammatory local cytokine response, we analyzed the cell populations in the lungs of uninfected and infected WT and PGLYRP2-KO mice for changes. Strikingly, we found fewer neutrophils in the lungs of infected PGLYRP2-KO compared to WT mice at 48 hpi. In fact, there was not only a reduction in PGLYRP2-KO mice but also a nearly abolished recruitment at this time compared to PBS-treated mice. This impaired immune response led to a worsened early control of bacterial spread and to a faster progression of the disease.

In our approximate survival analysis, we had to euthanize the PGLYRP2-KO mice 1.5 days earlier than the WT mice. This earlier euthanization was also correlated with higher clinical scores at earlier times in the PGLYRP2-KO mice. In contrast, there were no differences in the onset of clinical signs or the overall approximate survival in these experiments. However, one could imagine that a deficiency in *Pglyrp2* gene expression or function, e.g., a loss-of-function mutation by single nucleotide polymorphism (SNPs), could lead to higher mortality or worsened progression of pneumonia in humans because there would be less time for the treatment of ill patients. Unfortunately, there is little knowledge about SNPs in *Pglyrp2*. Only two reports suggested associations between SNPs in *Pglyrp2* and Parkinson’s disease (Goldman et al., 2014) or Crohn’s disease (Zulfiqar et al., 2013).

Still unresolved is why there was no difference in the overall approximate survival between the WT and PGLYRP2-KO mice. There are several possibilities that might explain these circumstances. First, neutrophil recruitment could be delayed instead of being completely abolished. Therefore, later recruitment would lead to the rescue of animals at later times. Second, neutrophil recruitment could be only impaired in the lungs. Recruitment into the blood might be different from what we see in the lungs. Third, the adaptive immune system might be unaffected by PGLYRP2 deficiency, and therefore neutralizing antibodies might rescue animals at approximately day 4–5. In addition, there might be several other mechanisms.

Taken together, we used the PGLYRP2-KO mouse model to analyze the function of this HDM in pneumococcal pneumonia. Deficiency of this particular gene led to early mortality of infected animals by an abolished neutrophil recruitment and increased spread of bacteria into the periphery. Nevertheless, animals, which survived the first 3–4 days, recovered from the infection. This is possibly mediated by protective, unaffected adaptive immune responses. However, we have no proof, so far, for this hypothesis. It is known that HDMs do not need to show direct antibacterial activity against a pathogen to affect the course of a disease (Hancock et al., 2016). Activation and modulation of major compartments of the innate immune system by those HDMs such as PGLYRP2 are alternative mechanisms to protect against diseases. Taking into account that neutrophils are the most necessary cell type to clear an infection and that their recruitment is impaired by a deficiency in PGLYRP2, one could imagine that polymorphisms in this gene could be directly linked to a higher susceptibility to pneumococcal pneumonia as well as other lung infections. Further studies should be conducted to decipher alterations of the immune system by PGLYRPs and possible implications for clinical usage.

## Author Contributions

AD, CC, UB, AS, NB, SW, and JZ performed the experiments. AD did formal analysis of the data and wrote the original draft. AD, CC, UB, AS, NB, SW, HH, SA, KR, NS, and JZ reviewed and edited the manuscript. SA, PN’G, KR, and JZ did conceptualization work. SA, KR, and JZ supervised the work. HH, SA, PN’G, and NS did funding acquisition. NS and JZ did project administration.

## Conflict of Interest Statement

The authors declare that the research was conducted in the absence of any commercial or financial relationships that could be construed as a potential conflict of interest.
