# Supplementary material for: Peptidoglycan Recognition Protein 2 Regulates Neutrophil Recruitment Into the Lungs After Streptococcus pneumoniae Infection
Source: Front Microbiol. 2019 Feb 19;10:199. doi: 10.3389/fmicb.2019.00199 (PMC6389715; doi:10.3389/fmicb.2019.00199)
Supplement: Supplementary file 1 [file Data_Sheet_1.pdf]

Supplementary Material

Peptidoglycan Recognition Protein 2 Regulates Neutrophil Recruitment into the Lungs After *Streptococcus pneumoniae* Infection

Alexander N. Dabrowski, Claudia Conrad, Ulrike Behrendt, Anshu Shrivastav, Nelli Baal, Sandra-M. Wienhold, Holger Hackstein, Philippe D. N'Guessan, Sahar Aly, Katrin Reppe, Norbert Suttorp and Janine Zahlten*

*** Correspondence:** Janine Zahlten: Janine.Zahlten@charite.de

Front. Microbiol. 10:199. doi: 10.3389/fmicb.2019.00199

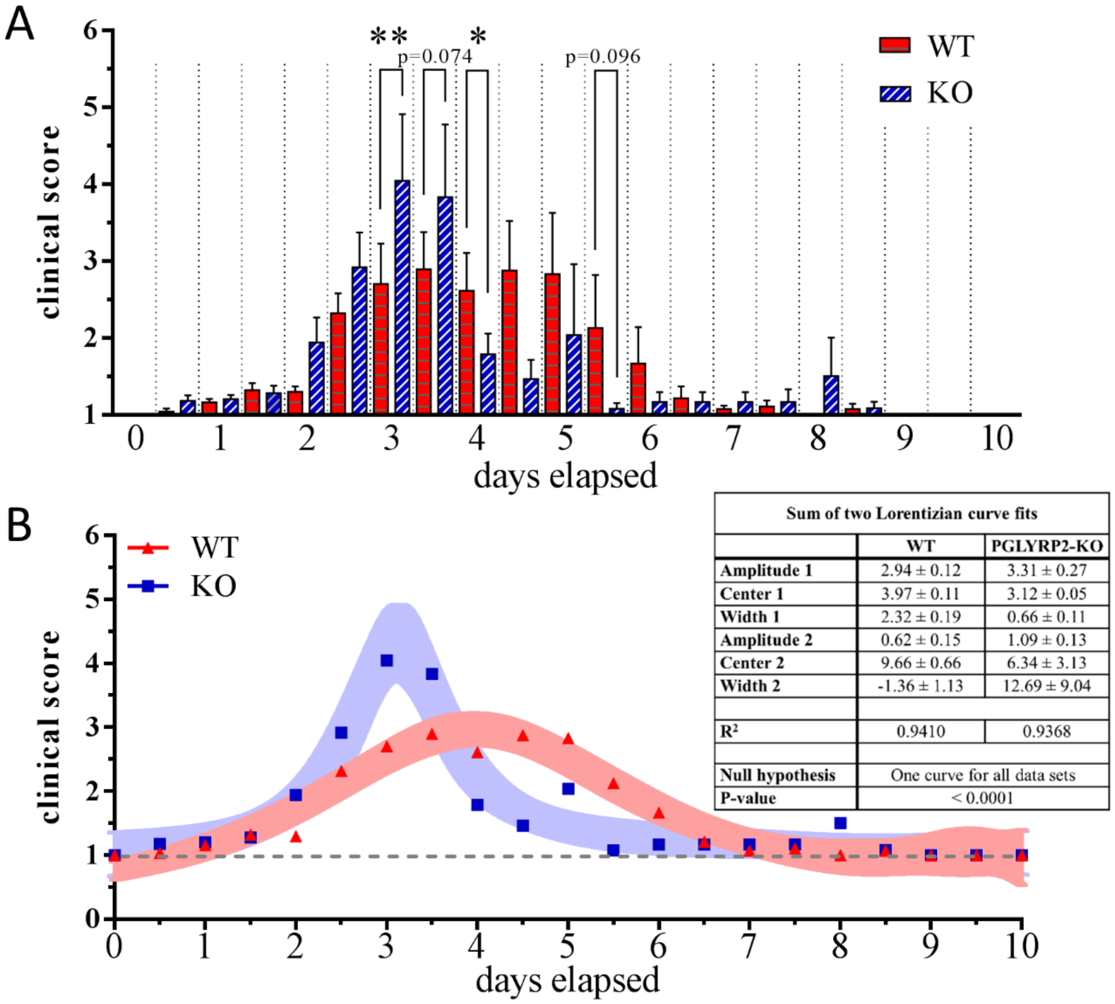

Figure S1: Clinical scores in the 10-day approximate survival experiment.
PGLYRP2-KO and WT mice infected with *S. pneumoniae* (A66, 5 x 10^4 CFU) were monitored for 10 days, and clinical signs for infection and sickness were recorded. **(A)** The clinical score was assessed and the means + SEMs are given (min. 1, max. 12). **(B)** The course of clinical scores was compared by a Lorentizian curve fit model. Dots represent the means of clinical scores, and bands represent the 95% CI of the curve fit with 25 (WT) and 24 (PGLYRP2-KO) mice. The grey dotted line represents the minimum of clinical score (1 = no signs of illness). Statistics: **(A)** Student's *t* test and **(B)** sum of two Lorentizian curve fits. * $p \leq 0.05$, ** $p \leq 0.01$, ns: not significant.

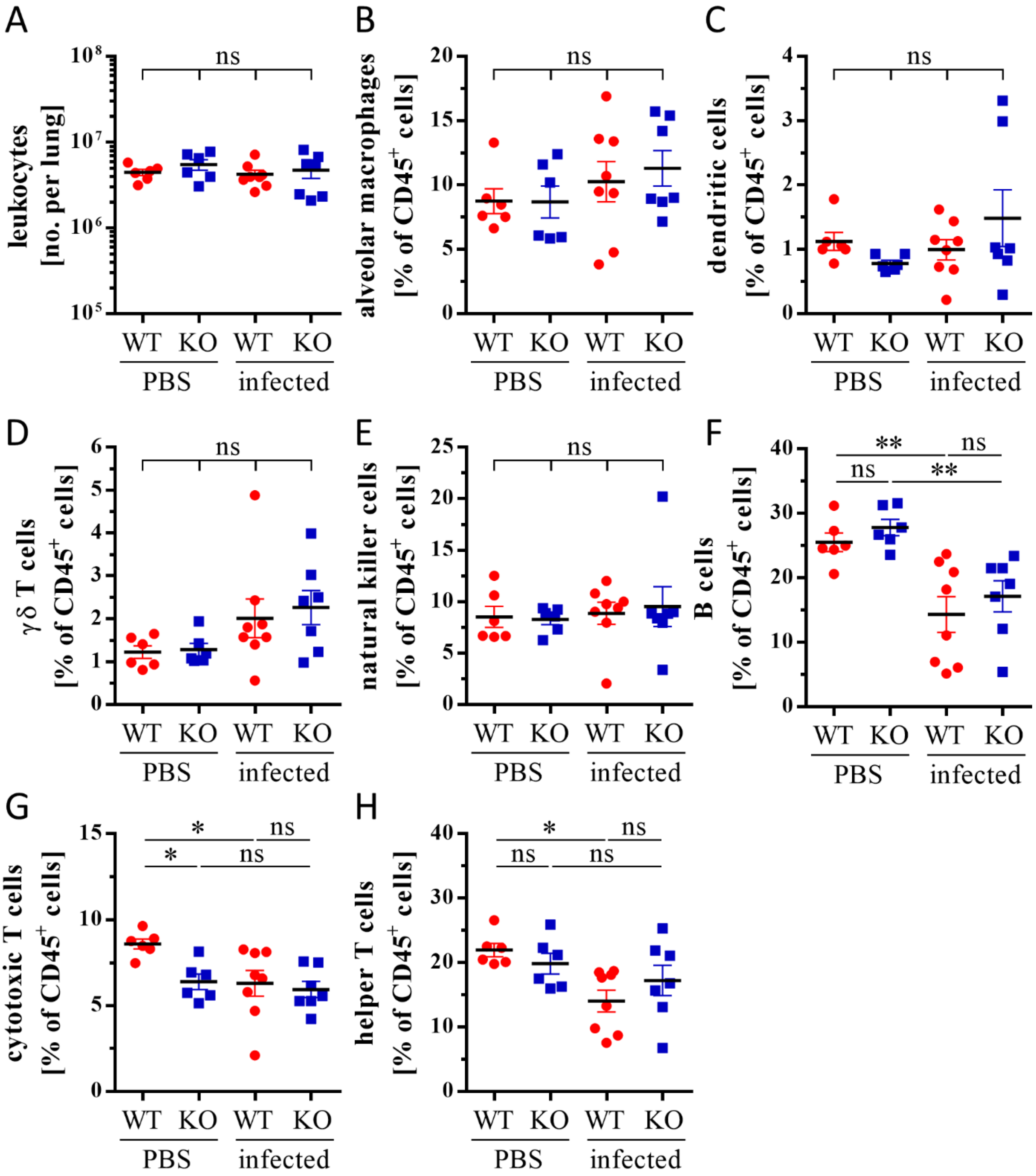

Figure S2: Cell recruitment into the lungs of WT and PGLYRP2-KO mice 48 hpi.
PBS and *S. pneumoniae*-infected animals (A66, 10^5 CFU) were analyzed 48 hpi. **(A)** Total number of leukocytes, **(B)** alveolar macrophages, **(C)** dendritic cells, **(D)** γδ T cells, **(E)** NK cells, **(F)** B cells, **(G)** cytotoxic T cells, and **(H)** helper T cells in the lungs were analyzed by flow cytometry. Means ± SEMs of six (WT PBS and PGLYRP2-KO), eight (WT infected) or seven (PGLYRP2-KO infected) samples. Statistics: ordinary one-way ANOVA with Holm-Sidak's correction for multiple comparisons. * $p \leq 0.05$, ** $p \leq 0.01$, ns: not significant.
